# Design and Optimization of a Novel SAW Gyroscope Structure Based on Amplitude Modulation with 1-D Phononic Crystals

**DOI:** 10.3390/mi12121485

**Published:** 2021-11-30

**Authors:** Fei Ge, Liye Zhao, Yang Zhang

**Affiliations:** School of Instrument Science and Engineering, Southeast University, Nanjing 210096, China; gefei96@163.com (F.G.); zhangy7597@163.com (Y.Z.)

**Keywords:** surface acoustic wave gyroscope, phononic crystals, traveling wave, amplitude modulation, theoretical modeling and simulation design

## Abstract

Surface acoustic wave gyroscopes (SAWGs), as a kind of all-solid-state micro-electro-mechanical system (MEMS) gyroscopes, can work normally under extremely high-impact environmental conditions. Among the current SAWGs, amplitude-modulated gyroscopes (AMGs) are all based on the same gyro effect, which was proved weak, and their sensitivity and intensity of the output are both lower than frequency-modulated gyroscopes (FMGs). However, because FMGs need to process a series of frequency signals, their signal processing and circuits are far less straightforward and simple than AMGs. In order to own both high-sensitivity and simple signal processing, a novel surface acoustic traveling wave gyroscope based on amplitude modulation is proposed, using one-dimensional phononic crystals (PCs) in this paper. In view of its specific structure, the proposed gyroscope consists of a surface acoustic wave oscillator and a surface acoustic wave delay line within a one-dimensional phononic crystal with a high-Q defect mode. In this paper, the working principle is analyzed theoretically through the partial wave method (PWM), and the gyroscopes with different numbers of PCs are also designed and studied by using the finite element method (FEM) and multiphysics simulation. The research results demonstrate that under a 1 V oscillator voltage output, the higher sensitivity of −23.1 mV·(rad/s)^−1^ in the linear range from −8 rad/s to 8 rad/s is reached when the gyro with three PC walls, and the wider linear range from −15 rad/s to 17.5 rad/s with the sensitivity of −6.7 mV·(rad/s)^−1^ with only one PC wall. Compared with the existing AMGs using metal dots to enhance the gyro effect, the sensitivity of the proposed gyro is increased by 15 to 112 times, and the linear range is increased by 4.6 to 186 times, even without the enhancement of the metal dots.

## 1. Introduction

As the core sensitive device of inertial navigation systems and inertial guidance systems, gyroscopes can simultaneously detect the angular velocity, angular acceleration, angular displacement and other information in multiple directions after being combined with an accelerometer to form an inertial measurement unit. Therefore, they have a wide range of applications in modern national defense construction and national economic construction. Among them, SAWGs, as a kind of typical all-solid-state MEMS gyroscopes, not only have the advantages of the general MEMS gyroscopes such as a small size, low cost, low power consumption, etc., but also contain no three-dimensional suspension vibration structures, for which they can work normally under an extremely high-impact environment. Therefore, they have a great demand in complex and extreme application fields [1,2].

SAWGs, according to the detected characteristic of a SAW, can be generally classified into two categories: amplitude-modulated gyroscopes (AMGs) and frequency-modulated gyroscopes (FMGs). The principle of AMGs is that, due to the Coriolis effect, the external angular velocity causes a metallic dot array, distributed inside the SAW resonator and vibrating with the standing wave, to excite the secondary SAW propagating perpendicularly to the propagation direction of the standing wave; then, it measures the angular velocity by detecting the amplitude of the secondary wave [3]. Since AMGs detect the amplitude and intensity of a SAW, one of its outstanding features is straightforward signal processing, no complicated signal modulation and a conversion process, only simple filtering and amplification. However, the sensitivity and intensity of the output signal of these kinds of gyros are really unsatisfactory; R. Clive Woods et al. questioned its sensitivity and estimated that its output voltage is only tens of nanovolts through more accurate calculations [4]. In order to improve the performance of AMGs, several methods had been tried: the modal coupling (COM) theory was used to optimized the SAW resonator that generated standing waves by Varadan et al., and the experimental result of 3.6 uV·(deg/s)^−1^ in the range of 10 deg/s was obtained [5]; the piezoelectric film, on which the metallic dot array was fabricated, was utilized to replace the traditional piezoelectric substrate by Lee’s research group. As a result, the amplitude of the standing wave in the cavity was increased, thereby the sensitivity of the gyro and the intensity of the output signal were improved, and a sensitivity of 27.5 uV·(deg/s)^−1^ was obtained in the range of 400 deg/s [6]. However, although the sensitivity of AMGs has somewhat improved through these efforts, it is still really low. Moreover, because what there is to detect is all weak secondary SAWs, the intensity of the output signal is only at a microvolt level.

FMGs were first proposed by Lee’s research group in response to the dilemma that the AMGs were subject to their own weak gyro effect. The principle is that the phase velocity of a SAW propagating on the piezoelectric medium changes due to the external angular velocity, thereby the center frequency of the SAW oscillator shifts. The prototype was created on the ST-cut quartz substrate, and the sensitivity was only 0.431 Hz·(deg/s)^−1^ in the range of 2000 deg/s [7]. Subsequently, in order to improve the sensitivity of these kinds of gyros, the research group cooperated with Weng Wang et al., adopting two methods successively: one method was coupling between the traveling wave and the secondary SAW generated by the SAW standing wave resonator [8,9,10], and the other was enhancing the Coriolis effect by adding a metallic dot array inside the SAW traveling wave oscillator [11,12,13]. FMGs have had a significant improvement in sensitivity compared to AMGs and, at the same time, have overcome the fatal shortcoming of AMGs not being able to compensate for temperature drifts. However, FMGs have a more complicated signal processing process compared to AMGs: the two output signals need to be filtered and mixed to obtain a low-frequency offset signal, and then the frequency offset signal is converted into a pulse signal after an amplification and shaping circuit, and the final result is obtained after the pulse signal is processed cooperatively by a timing control circuit, a gate control circuit and a counter.

In recent years, PCs, with which sound waves and elastic waves could be manipulated artificially, have been widely applied in sensing [14,15], waveguide [16], filtering [17,18] and other fields. One of the most important characteristics of PCs is the defect mode, and a high-Q defect mode can be obtained by rationally selecting materials and designing a structure [17,19,20,21,22]. This high-Q defect mode is extremely sensitive to the frequency of the excitation signal, and can be utilized in sensors to obtain an exceedingly high sensitivity and resolution, thereby providing an inspiration for novel SAWGs.

In order to retain the advantages of simple signal acquisition and the processing of AMGs while combining the advantages of the high sensitivity of FMGs, this paper proposes an amplitude-modulated surface acoustic traveling wave gyroscope based on one-dimensional PCs. Additionally, the proposed AMGs ultimately detect the first surface acoustic traveling wave, which has a higher amplitude, instead of the secondary SAW. The structure of this paper is arranged as follows: In the second section, the detailed design of the structure and the theoretical analysis of this gyro are provided, respectively. Then, in the third section, the gyro, including its components, is optimized, and the performance of the optimized gyro is also analyzed. Finally, we provide a conclusion in the fourth section.

## 2. Design and Theory

### 2.1. Design of Basic Structure and Working Principle

**Figure 1:**
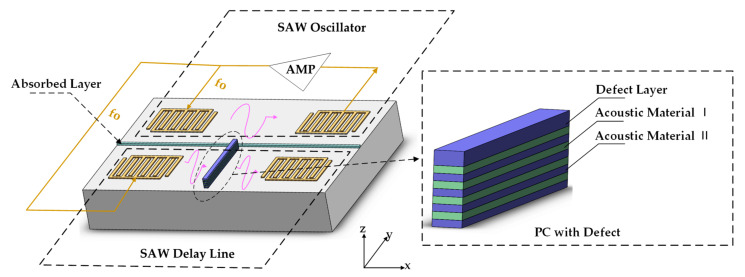
Structure of the proposed SAWGs. The gyro was composed of a SAW oscillator and a SAW delay line with one-dimensional PCs in the insert.

As shown in Figure 1, the sensitive component of the phononic crystal-based SAWG proposed in this article was composed of two parts: a SAW oscillator and a SAW delay line with PCs inside. The SAW oscillator consisted of a SAW delay line and an external feedback amplifier and, in the delay line, the excitation interdigital transducers (IDTs) were connected with the receiving IDTs through the feedback amplifier to form a self-oscillation circuit. The delay line containing the PCs inside was composed of another delay line and a SAW modulation structure placed inside the delay line, and the essence of the SAW modulation structure was one-dimensional PCs alternately stacked by two kinds of phononic materials along the z axis direction and containing a defect layer. When the defect mode introduced by the defect layer fell within the band gap of the PCs, the SAW, whose frequency was within the band gap but did not match the defect mode, would not be coupled into the PCs, and so could spread to the receiving IDTs unaffected; the SAW whose frequency matched the defect mode would be absorbed by the PCs, so the output signal of the received IDTs appeared as an attenuation of intensity.

**Figure 2:**
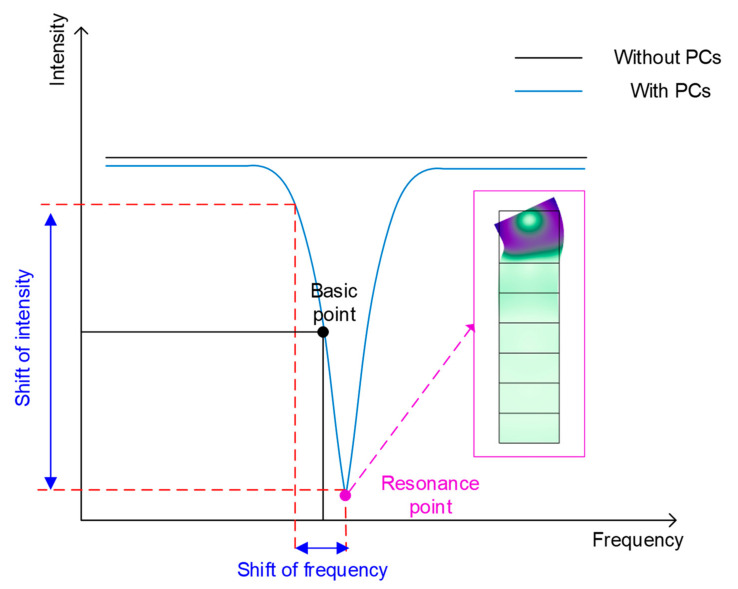
Working principle of the proposed SAWGs.

When the gyro worked, as shown in Figure 2, the initial center oscillation frequency of the SAW oscillator was arranged at the basic point, where the slope on the side of the resonance valley of the PCs defect mode was steepest and the absorption of the SAW by PCs was most sensitive to the shift of frequency. If there was an angular velocity input around the y axis, the center oscillation frequency of the SAW oscillator shifted accordingly. Since the output terminal of the oscillator was additionally led out as the excitation of the SAW delay line, the frequency of the SAW in the delay line also changed with the external angular velocity at the same as the SAW oscillator, and then this frequency change would be strongly modulated by the PCs in the defect mode. At the same time, the SAW amplitude itself would also be affected by the angular velocity, so the intensity of the output signal would be modulated by two aspects at the same time.

### 2.2. Theoretical Analysis

Consider an anisotropic and piezoelectric medium occupying a half-space $\left(x_{3}\leq 0\right)$ with no mechanical load about the plane $\left(x_{3}=0\right)$ and rotating at a constant angular rate $\left(\Omega_{i}\right)$ about the $x_{i}$ axis (i = 1, 2, 3). The dynamic equations coupling with the contribution of the Coriolis effect and centrifugal forces could be expressed as:

(1) $$\left\{\begin{array}{l} c_{ijkl}^{E}\frac{\partial^{2}u_{k}}{\partial x_{j}\partial x_{i}}+e_{kij}\frac{\partial^{2}\varphi }{\partial x_{k}\partial x_{j}}=\rho \left[\frac{\partial^{2}u_{i}}{\partial t^{2}}+2\varepsilon_{ijk}\Omega_{j}\frac{\partial u_{k}}{\partial t}-\left(\Omega_{j}{}^{2}u_{i}-\Omega_{i}\Omega_{j}u_{j}\right)\right]\\ e_{ikl}\frac{\partial^{2}u_{k}}{\partial x_{l}\partial x_{i}}-\varepsilon_{ik}^{S}\frac{\partial^{2}\varphi }{\partial x_{k}\partial x_{i}}=0 \end{array}\right.i,j,k,l=1,2,3$$

where $c_{ijkl}^{E}$, $e_{kij}$, and $\varepsilon_{ik}^{S}$ are the elastic stiffness constant when the electric field strength is zero or constant, the piezoelectric stress constant, and the dielectric constant when the strain is zero or constant, respectively. The displacement of the particle and the electric potential are denoted by $u_{i}$ and φ, respectively. ρ stands for the density of the medium and $\varepsilon_{ijk}$ is the Levi-Civita symbol.

In this paper, the PWM [23] was utilized to solve the dynamic coupled equations, whose solution was regarded as a linear superposition of different waves, so we could preset the form of the solution to bring back into the dynamic coupled equations and, finally, the correctness of the solution was verified by substituting the boundary conditions. The 128° YX LiNbO_3_, whose material parameters were referred to by [24], was selected as the piezoelectric substrate because of its relatively high electromechanical coupling coefficient (*K*^2^ = 5.56%).

**Figure 3:**
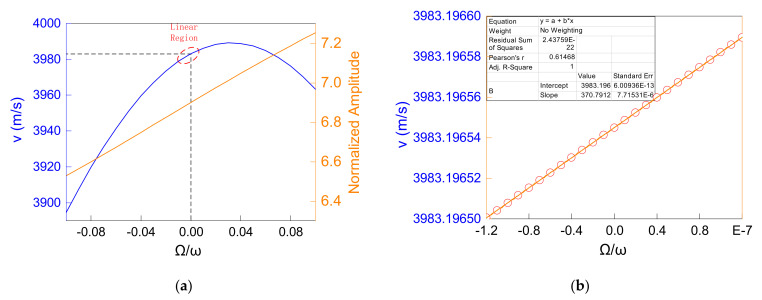
Calculation results of the dynamic coupled equations by utilizing the FWM: (**a**) the trend of phase velocity and normalized amplitude under the ratio of rotation speed ranging from −0.1 to 0.1; (**b**) the trend of phase velocity under a small range near the zero angular velocity point.

After the calculation, when the substrate rotated around the $x_{2}$ axis, the relationship between the angular velocity and the properties of the SAW propagating along the $x_{1}$ axis, such as the phase velocity and amplitude, is shown in Figure 3. Figure 3a suggests that the normalized amplitude of SAW had an obvious linear relationship with an angular velocity under a wide range of angular velocity changes, while its phase velocity and angular velocity had an asymmetric quadratic relationship. Moreover, in Figure 3b, it is worth noting that the phase velocity also had a linear relationship with the angular velocity in a small range near the zero angular velocity point, which was also the working range of the gyroscope in this paper. The relationship between the phase velocity, normalized surface wave amplitude and angular velocity in this working range could be expressed as:

(2) $$\left\{\begin{array}{l} v\left(\gamma \right)=\left.\frac{\partial v\left(\gamma \right)}{\partial \gamma }\right|\cdot \gamma +v_{0}=K_{v}\cdot \gamma +v_{0}=\Delta v\left(\gamma \right)+v_{0}\\ \left|u\left(\gamma \right)\right|=\sqrt{\sum_{i=1}^{3}u_{i}{\left(\gamma \right)}^{2}}=K_{amp}\cdot \gamma +u_{0} \end{array}\right.,$$

where γ is denoted as the ratio of rotation speed Ω/ω, ω is the angular frequency of SAW, $v_{0}$ and $u_{0}$ are the phase velocity and the normalized SAW amplitude at zero angular velocity, respectively, and $K_{v}$ and $K_{amp}$ are defined as the phase velocity gain coefficient and amplitude gain coefficient, respectively. Due to the high linearity in the working range of the gyro, this representation was obviously reasonable.

In a SAW oscillator with a delay line length of L and a SAW wavelength of $\lambda \left(L\gg \lambda \right)$, besides satisfying the loss compensation condition, it also had to satisfy the phase-matching condition:

(3) 2πfL/v=2nπ,

where *f* is the frequency of SAW in the delay line and is also the oscillation frequency of the oscillator, and n is a positive integer. When the angular velocity was input, f would also shift due to the change of SAW’s phase velocity:

(4) $$\Delta f=f\left(\gamma \right)-f_{0}=\frac{n}{L}\left[v\left(\gamma \right)-v_{0}\right]=\frac{n}{L}\cdot K_{v}\cdot \lambda ,$$

when L is an integer multiple of λ, the frequency shift of the center frequency of the oscillator could be simplified as:

(5) $$\Delta f=\Delta v\left(\gamma \right)/\lambda ,$$

when the initial frequency $f_{0}$ of the SAW without an angular velocity was at the basic point, since the frequency shift produced by the external angular velocity would have a nearly linear and significant impact on the amplitude of the SAW, the amplitude change caused by the frequency shift could be expressed as:

(6) $$\Delta \left|A_{freq}\right|=K_{fPC}\cdot \Delta f,$$

where $K_{fPC}$ is the amplitude gain coefficient derived from the frequency shift effect of PCs. However, on a free surface without PCs, the angular velocity itself would also affect the amplitude of SAW:

(7) $$\Delta \left|A_{\Omega }\right|=\frac{\left|A\right|}{u_{0}}\cdot \Delta \left|u_{3}\left(\gamma \right)\right|,$$

Therefore, when the SAW passed through the PCs, if there was an external angular velocity input, on the one hand, the amplitude of the SAW was modulated by the PCs’ frequency shift effect and, on the other hand, it was directly affected by the angular velocity on the amplitude, so that the amplitude of the SAW after the PCs changed as:

(8) $$\begin{array}{l} \Delta \left|A\right|=\Delta \left|A_{freq}\right|+\left|\frac{A_{0}+\Delta \left|A_{freq}\right|}{u_{0}}\right|\cdot \Delta \left|u\left(\gamma \right)\right|\\ =\left(\frac{K_{fPC}K_{v}}{\lambda }+\frac{K_{amp}\left|A_{0}\right|}{u_{0}}\right)\gamma +\frac{K_{fPC}K_{v}K_{amp}}{\lambda }\gamma^{2} \end{array},$$

where $A_{0}$ is the SAW amplitude at the basic point with no angular velocity. Since the gyro worked at a very small ratio of rotation speed γ, the quadratic term of the above formula could be ignored. Therefore, the modulation of the amplitude of SAW by PCs could be approximated as a linear superposition of the two effects of amplitude change caused by the frequency shift under the modulation of PCs and the amplitude change caused by the angular velocity on the free surface. According to the theory in [25], the change of voltage output of IDTs could be obtained as:

(9) $$\Delta V_{out}=4\omega NW\left[JLG\left(\Delta \right)F\left(k_{s}\right)\right]Z_{t}\cdot \Delta \left|A\right|,$$

where N and W are the number and length of the received IDTs, respectively; J and L are the flux constant and amplitude constant, respectively, both of which are determined by the material; $G\left(\Delta \right)$ is determined by the geometry of the transducers; $F\left(k_{s}\right)$ depends on the frequency of the external excitation; $Z_{t}$ is the impedance of the transducers. It can be seen that the output voltage of the transducers and the SAW amplitude were also linear, so the sensitivity S of the gyro could be expressed as:

(10) $$\begin{array}{l} S=\frac{\Delta V_{out}}{\Omega }=4NW\left[JLG\left(\Delta \right)F\left(k_{s}\right)\right]Z_{t}\cdot \left(\frac{K_{fPC}K_{v}}{\lambda }+\frac{K_{amp}\left|A_{0}\right|}{u_{0}}\right)\\ =K_{e}\cdot \left(\frac{K_{fPC}K_{v}}{\lambda }+\frac{K_{amp}\left|A_{0}\right|}{u_{0}}\right) \end{array},$$

under a fixed material, temperature and excitation conditions: $K_{v}$, $K_{amp}$, $\left|A_{0}\right|$ and $u_{0}$ are constants; $K_{e}$ is related to the structure of the transducers, the lower the transducer loss, the greater the $K_{e}$; $K_{fPC}$ is related to the structure of PCs. Therefore, the design of PCs and IDTs should have been considered in the process of designing the gyro.

## 3. Optimization and Analysis

### 3.1. Optimization and Analysis of PCs

**Figure 4:**
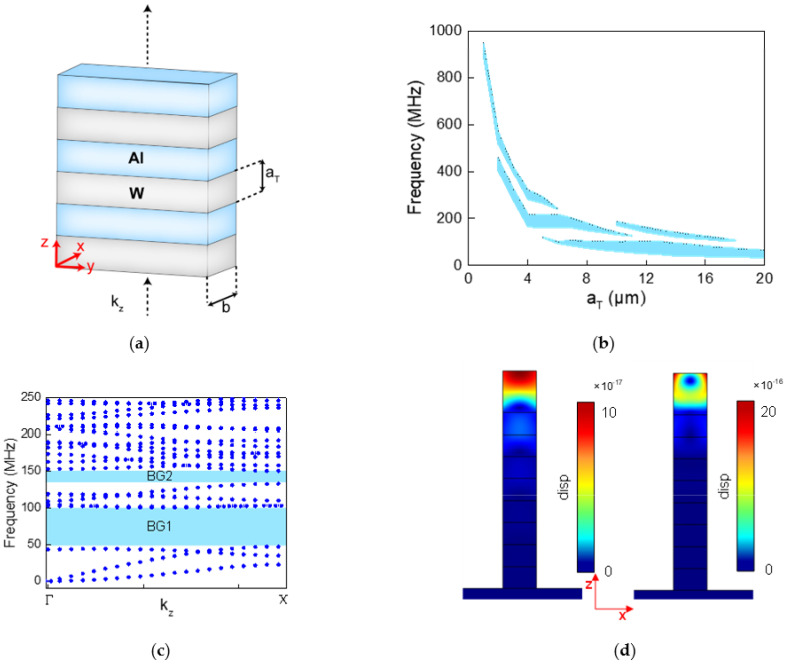
(**a**) Basic structure of the one-dimensional PCs used in the proposed SAWG; (**b**) distribution of band gaps as $\alpha_{T}$ ranged from 2 to 20 μm; (**c**) band diagram of PCs in the Brillouin zone when $\alpha_{T}$ = 13 μm; (**d**) defect modes existing in the BG1 of (**b**): the left one is compressional mode and the right one is flexural mode.

The structure of the one-dimensional PC used in the proposed SAWG is shown in Figure 4a, which was composed of two phononic material layers stacked alternately along the Z axis [21]. Each phononic material layer extended infinitely along the Y axis, having the same thickness of $\alpha_{T}$ along the Z axis and width b along the X axis. Therefore, the lattice period of the PCs was $T=2\alpha_{T}$. The phononic materials selected were tungsten and aluminum, which have a large density difference and elastic modulus difference between one another, in order to obtain a wider complete band gap.

The distribution of the PC band gaps was mainly related to the structural parameter $\alpha_{T}$, while b only had a slight influence on it. However, with the growth of b, the loss of SAW after PCs would increase and the aspect ratio of the structure of PCs would decrease; therefore, b was always selected as 20 μm in this paper under comprehensive consideration. The COMSOL Multiphysics software (version 5.5) was utilized to calculate the band structure of the PCs along the Z axis with different thickness of $\alpha_{T}$, and the relationship between the band gaps and $\alpha_{T}$ was obtained as shown in Figure 4b. When the lattice period of the one-dimensional PC was small, the band gap was distributed in the high frequency band, and the band gap range was narrow, which was not conducive to the design of IDTs and the introduction of defect states in the PC. The range of the band gaps moved from a high frequency to low frequency with the increase in $\alpha_{T}$, and each $\alpha_{T}$ could have corresponded to one or more band gaps at the same time. The widest band gap appeared in the lower-frequency area when $\alpha_{T}$ was 13 μm. Figure 4c shows the band diagram of the PCs in the Brillouin zone when $\alpha_{T}$ = 13 μm, and there was an obvious band gap BG1 in the range of 47 to 100 MHz. Then, even if $\alpha_{T}$ continued to grow, the band gaps would only move down slightly, so 13 μm was chosen as the thickness of each layer of phononic material.

The defect mode could be introduced by adding an extra layer of Al with a thickness of e as the defect layer on top of the eight-layer PC. There were two defect modes in the band gap BG1, as shown in Figure 4d, the left one was the compressional mode, where the defect layer was deformed along the Z axis, and the other was the flexural mode, where the defect layer was bent out of the YZ plane. The common feature of these two defect modes was that the deformation and stress were concentrated in the defect layer on the top of the PC, while the other PC layers outside the defect layer were almost stress-free, which appeared as that when the SAW passed through the PCs, and the energy was strongly coupled into the top of the PCs to resonate, and the amplitude and intensity of the SAW was greatly attenuated. Comparing these two defect modes, the strain at the top of the flexural mode was about 20 times higher than that of the compressional mode, which showed that the flexural mode could limit more of the SAW’s energy; therefore, the flexural mode was selected as the working mode of the gyro.

**Figure 5:**
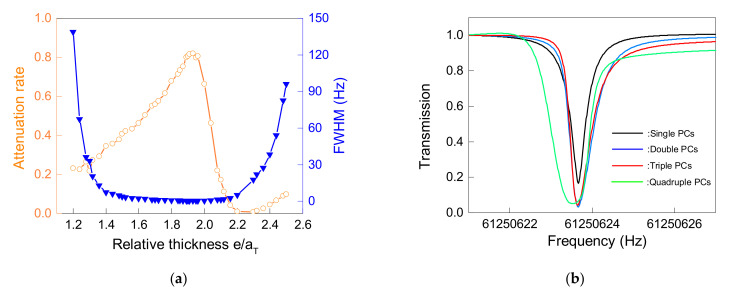
(**a**) Relationship between the relative thickness of the defect layer and the properties of the resonant peak: the orange curve stands for attenuation rate and the blue curve stands for FWHM; (**b**) transmission curve of PCs with incremental walls (1 to 4 walls).

For a single PC wall, $K_{fPC}$ was mainly related to the energy attenuation rate of the SAW in the defect mode and the full width of the half maximum (FWHM) of the resonance peak. By adjusting the size of e, these two parameters in the defect mode could be controlled, thereby adjusting $K_{fPC}$. Figure 5a shows the effect of e on the energy attenuation rate and FWHM of the defect mode: the energy attenuation rate increased first and then decreased as the thickness of e increased, and had a maximum value near the relative thickness of 1.9; the FWHM had a tendency to decrease first and then increase, and had a bandwidth narrower than 1 Hz in a wide range of the relative thickness from 1.6 to 2.0. To increase $K_{fPC}$, it was not only necessary to increase the attenuation rate, but also to reduce the FWHM. Therefore, the relative thickness of the defect layer was selected as 1.91 and, at this time, the attenuation rate was as high as 0.82, while the FWHM was as narrow as 0.4 Hz.

Moreover, increasing the number of PC walls along the *X* axis would also have had a complex impact on $K_{fPC}$. Figure 5b shows the relationship between increasing the number of PC walls and the transmission rate of the SAW at the defect mode. As the number of walls increased, the transmission rate of the SAW by PCs would decrease, which was beneficial to the improvement of $K_{fPC}$. However, in the process from the single wall to double walls, the transmission rate of the SAW was decreased from 0.18 to 0.045, while the triple walls only decreased to 0.03, and then even if the number of walls increased, it could only be decreased to a limited extent. At the same time, the process of increasing the number of walls would broaden the FWHM of the resonance peak, which was not conducive to the improvement of $K_{fPC}$. These phenomena were caused by the introduction of degenerate modes, the number of which was equal to the number of walls. Since the absorption of SAW by PCs at the defect mode was the superposition of several degenerate modes, and there were small frequency differences among these degenerate modes, as the number of walls increased, the SAW transmission rate decreased and the FWHM widened. Additionally, after the number of PCs increased to four or more, the decrease in the transmission rate of the SAW would be very limited, while the FWHM would continue to widen; therefore, even if it continued to increase the number of PCs, $K_{fPC}$ could not always increase and the number of PCs could reach the highest $K_{fPC}$ within four layers. Based on the above design and analysis, the design parameters of PCs can be summarized in Table 1.

### 3.2. Optimization and Analysis of IDTs

The IDTs constituted two SAW delay lines in the gyroscope in this paper: one was connected with the feedback amplifier to form a SAW oscillator, and the other used the oscillator output as an excitation. IDTs with a low loss were beneficial to increase $K_{e}$, thereby increasing the sensitivity of the gyro. The IDTs in this paper chose single-phase unidirectional transducers (SPUDTs), which could not only suppress the three-time crossing signal through the internal reflection of the transducer, but could also effectively reduce the two-way loss of the transducer due to its inherent unidirectionality, thereby improving the insertion loss and increasing $K_{e}$ [26].

**Figure 6:**
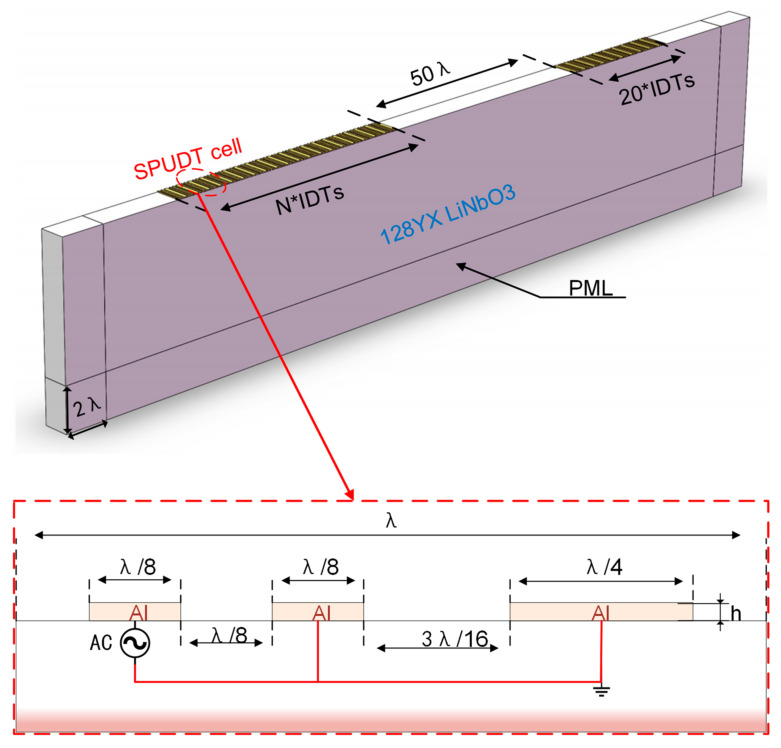
Simulation model of the SAW delay line built in COMSOL Multiphysics software. Structure of single-cycle SPUDT and its terminal boundary conditions are shown in the insert.

We built the model as shown in Figure 6, and the performance of the delay line based on SPUDTs could be predicted accurately and fast by the FEW following the steps in [27]. The delay line structure was constructed on the surface of the 128° YX LiNbO_3_ substrate with aluminum as the electrodes. The two sides and the bottom of the substrate had a perfectly matched layer with a thickness of two times the interdigital period of 2 λ to suppress the reflection of the wave at the boundary. Additionally, and the continuity periodic boundary conditions were added to the two sides with the y axis as the normal direction to equivalently extend infinitely in the y axis direction. A single-cycle SPUDT with the length of λ contained three electrodes, of which the thickness was set to h/λ=0.6%, and the leftmost electrode was connected to an AC power source, while the remaining electrodes were grounded. In the delay line, there were N pairs of excitation IDTs and 20 pairs of reception IDTs distributed, between which the distance was set to 50 times the interdigital period of 50 λ.

**Figure 7:**
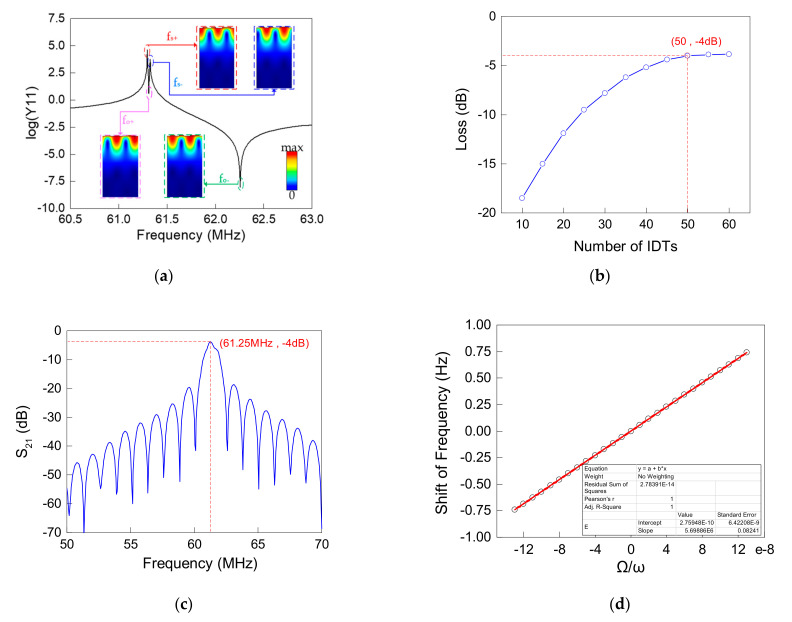
(**a**) Admittance curve of SPUDTs when λ = 64.3 μm with the mode shape of each mode in the insert; (**b**) relationship between insertion loss of the SAW delay line and the number of excitation IDTs; (**c**) S_21_ parameter (response in frequency domain) of the SAW delay line with 50 pairs of excitation IDTs; (**d**) relationship between the shift of center frequency of the SAW oscillator based on the designed SAW delay line and the ratio of the rotation speed ranging from −1.3 × 10^−7^ to 1.3 × 10^−7^.

The working frequency of SPUDTs needed to match the frequency of the defect mode of PCs, so that the gyro could work normally, which could be achieved by adjusting the length λ of a single cycle. Figure 7a shows the admittance curve of SPUDTs, each pole in the curve corresponding intuitively to a certain mode, where fo+ and fo− refer to the resonant frequency and anti-resonant frequency of the SPUDTs in the open state (free charge boundary condition applied to the leftmost electrode). In the ideal state, when the frequency was matched, the frequency of the defect mode, the resonant frequency fo+ and the characteristic frequency fs of the SPUDTs in the short-circuit state (the leftmost electrode was connected to the power supply working state), were equal to each other. However, due to the reflection effect of the periodic electrodes, the characteristic frequency in the short-circuit state was split into fs− and fs+, and the average value of fs+ and fs− could be considered as the characteristic frequency fs. According to the admittance curve, when the frequency was matched, the single-cycle length λ of SPUDT was 64.3 μm.

In addition to optimizing the structure of single-period IDTs, the $K_{e}$ could also be improved by increasing the number of electrodes. Figure 7b shows the relationship between the number of IDTs and the delay line loss and indicating that, as the number of IDTs increased, the loss of the delay line continued to decrease with the trend slowing down, and the loss of delay line stabilized at −4 dB when the number of IDTs increased to 50. At this time, the frequency domain response of the delay line is shown in Figure 7c, with the lowest loss of −4 dB at 61.25 MHz. The basic point of the gyro was always slightly biased to the frequency of the defect mode, so the loss in the actual work was slightly higher than −4 dB, but the delay line was still applicable. Based on the above analysis, the design parameters of the delay line could be summarized as Table 2. Based on the delay line and the PWM (mentioned and analyzed in the second section), the relationship between the shift of the center frequency of the oscillator and the angular velocity along the y axis is shown in Figure 7d. In the SAW oscillator that did not consider the enhanced gyro effect from the metallic lattice, every 1 × 10^−7^ normalized angular velocity could produce a frequency shift of 0.58 Hz.

### 3.3. Comprehensive Analysis

**Figure 8:**
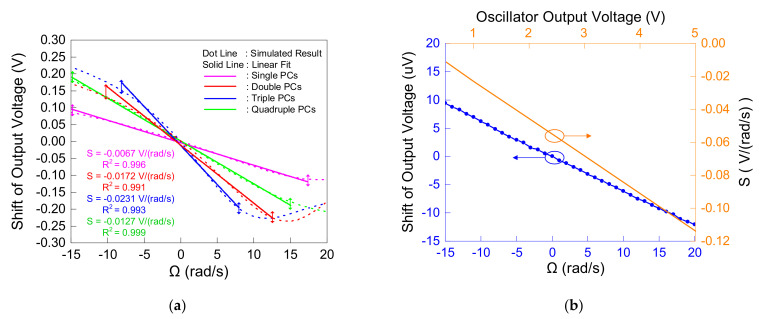
(**a**) Relationship between angular velocity around *Y* axis and shift of output voltage of gyros with 1 to 4 PC walls, respectively; (**b**) blue curve: shift of output voltage with varied angular velocity around *Y* axis when not considering the modulation of PCs for frequency shift; orange curve: sensitivity of proposed gyros with varied oscillator output.

The gyro structure with different numbers (range from one to four) of PC walls was simulated and analyzed, respectively. Under the steady amplitude voltage output of the SAW oscillator of 1 V, the relationship between the output voltage shift of the four kinds of gyros and the angular velocity input around the y axis is shown in Figure 8a. For the gyro with only one PC wall, a good linear response was obtained in the range of −15 rad/s to 17.5 rad/s. The slope of the straight line obtained from the linear fitting represented the shift of the output voltage under a per unit angular velocity input, which was equivalent to the sensitivity S. Therefore, the sensitivity of the gyro with a single PC wall was −0.0067 V·(rad/s)^−1^. When the number of PC walls was increased to two, the sensitivity was also increased to −0.0172 V·(rad/s)^−1^, which benefited from the double absorption of the SAW by the double PC walls. However, while the sensitivity was improved, the linear range was shortened from −10 to 12.5 rad/s. With triple PC walls, the sensitivity was further improved to −0.0231 V·(rad/s)^−1^, while the linear range was further reduced, and ranged from −8 to 8 rad/s; meanwhile, the sensitivity reached the highest value. Then, when the number of walls was increased to four, since the attenuation effect on the sensitivity from degenerate modes was greater than the improvement effect on the sensitivity from the multiple absorption of the PC walls, the sensitivity was greatly reduced to −0.0127 V·(rad/s)^−1^ and, relatively, the linear range was extended to −15 to 15 rad/s.

In addition, the result shown in Figure 8a was the superposition of the SAW amplitude change caused by the PC modulation by the frequency shift and the SAW amplitude change caused by the angular velocity. When only the latter was considered, that is, the delay line containing the PCs in the gyro used a fixed-frequency alternating power supply instead of the output voltage of the oscillator as an excitation, the result is shown by the blue curve in Figure 8b. Although the change of the amplitude of the SAW was still nearly linear, the change in the output voltage was only in the order of microvolts, which was nearly four orders of magnitude away from the output change, considering both cases at the same time. Therefore, the influence of the latter could be ignored, and it was considered that the output of the gyro was only determined by the modulation of the PCs on the frequency shift.

At the same time, the orange curve in Figure 8b took the gyro with triple PC walls as an example to show the relationship between the amplitude of the output voltage of the oscillator and the sensitivity of the gyro. The sensitivity of the gyro would be increased proportionally by increasing the output voltage of the oscillator. This was because increasing the voltage amplitude would enhance the intensity and amplitude of the SAW excited by it, while the proportion of the SAW absorbed into the PCs remained unchanged, resulting in more energy absorption and a greater output change.

Finally, the gyro proposed in this paper was compared and analyzed with the existing SAW gyros in Table 3. Compared with the existing AMGs, although the proposed gyro did not contain a metallic array to increase the gyro effect, the sensitivity and intensity of the output signal and linear range were significantly improved. These improvements, on the one hand, resulted from the more sensitive gyro effect than traditional AMGs’; one the other hand, they benefited from detecting the primary traveling wave, which was stronger than the secondary SAW detected by existing AMGs. Additionally, compared with the existing FMGs, the linear range of this gyro was reduced, while the signal processing circuit was relatively simpler. This was because the structure of the high-Q PCs was equivalent to a modulation amplifier, which amplified the tiny frequency shift and modulated it into an analog voltage output within the structure; thus, saving the original mixing, shaping and frequency detection circuits. However, the high-Q defect mode had a narrow FWHM, which reduced the linear range.

## 4. Conclusions

In this paper, an amplitude-modulated surface acoustic traveling wave gyroscope based on one-dimensional PCs was proposed, and the principle, structure and performance of the gyroscope were researched and analyzed by using the FEM and PWM. The one-dimensional PCs with a high-Q defect mode were very sensitive to frequency shifts, and a tiny frequency shift could produce a large SAW amplitude change, which was equivalent to a modulation amplifier. Moreover, the primary SAW traveling wave had a higher amplitude than the secondary SAW excited by the standing wave under the Coriolis effect, and a stronger output signal could be obtained.

Through a simulation and analysis, under the 1 V oscillator output, as the number of PC walls in the gyro increased from one to four, the sensitivity of the gyro also changed in the range of −0.0067 V·(rad/s)^−1^ to −0.0231 V·(rad/s)^−1^, and when the number of PC walls was three, it had the highest sensitivity, and the linear range was from −8 to 8 rad/s. Meanwhile, the sensitivity could be further improved by increasing the output of the oscillator. Compared with the existing SAW gyroscopes, the output intensity and sensitivity of the gyroscope were improved.

## Figures and Tables

**Table 1:** Design parameters of PCs.

Parameter	Value
Phononic material	Al and W
Thickness of single-layer phononic material (a_T_)	13 μm
Width of PCs (b)	20 μm
Material of defect layer	Al
Relative thickness of defect layer (e/a_T_)	1.91
Number of PC walls	1~4

**Table 2:** Design parameters of the SAW delay line.

Parameter	Value
Substrate material	128° XY LiNbO_3_
Electrode material	Al
Period of SPUDTs (λ)	64.3 μm
Number of excitation electrodes	50
Number of receiving electrodes	20
Length of delay line	50 λ
Operating frequency	61.25 MHz
Insertion loss	−4 dB

**Table 3:** Comparison of the proposed SAWGs with representative AMGs and FMGs.

Performance and Characteristic	The Proposed Gyro	Amplitude-Modulated		Frequency-Modulated	
		[4]	[6]	[7]	[11]
Sensitivity	23.1 mV/(rad/s) under 1 V	3.6 uV/(deg/s)	27.5 uV/(deg/s) under 3.7 V	0.431 Hz/(deg/s)	62.57 Hz/(deg/s)
Linearity range	±8 rad/s	0 to 10 deg/s	0 to 400 deg/s	0 to 2000 deg/s	0 to 1000 deg/s
Operating frequency	61.25 MHz	75 MHz	160 MHz	98.6 MHz	80 MHz
Metallic array	No	Yes	Yes	No	Yes
Signal processing circuit	Simple	Simple	Simple	Complex	Complex
